# Independent AI pipelines for rehabilitation exercise assessment and cognitive status classification: a retrospective proof of concept study

**DOI:** 10.3389/fpubh.2026.1951198

**Published:** 2026-10-14

**Authors:** Guoyu Wang, Guoliang Ren, Jiaju Zhu

**Affiliations:** 1 Department of Physical Education, Graduate School, Pukyong National University, Busan, Republic of Korea; 2 Graduate School, Jilin Sport University, Changchun, Jilin, China; 3 Shenzhen Institutes of Advanced Technology, Chinese Academy of Sciences, Shenzhen, China

**Keywords:** artificial intelligence, cognitive impairment, digital health, exercise assessment, explainable machine learning, healthy aging, older adults, telerehabilitation

## Abstract

**Background:**

Digital rehabilitation can extend remote exercise monitoring and cognitive health support, but available public datasets often capture these functions separately. This study evaluates two independent machine learning pipelines within a conceptual telerehabilitation architecture rather than a clinically validated integrated system.

**Methods:**

The physical pipeline used the WLU Rehabilitation Posture dataset. After quality control and removal of exact duplicates, we retained 315 unique videos of arm raise, knee extension, and sit-to-stand with dataset-defined correct or incorrect labels. We converted 24 video frames into 182 privacy-preserving features: appearance, shape, gradient, optical flow, temporal, and metadata. We compared candidate algorithms using stratified five-fold cross-validation within the training data, reserving a 20% holdout set for final evaluation. The cognitive pipeline analyzed 5,000 records from the Community Elderly Cognitive Health dataset using 14 demographic, clinical, lifestyle, psychological, and cognitive-assessment predictors. Because target generation is not independently documented and MMSE and GDS are target-proximal, this module is interpreted as concurrent cognitive status classification rather than as independent risk prediction.

**Results:**

Random forest achieved video-level physical six-class accuracy of 0.857, balanced accuracy of 0.846, macro F1 of 0.849, and Matthews correlation coefficient of 0.829; because participant and session identifiers were unavailable, these estimates do not represent participant-independent validation. Exercise identity accuracy was 0.984, whereas correct versus incorrect execution accuracy was 0.857. Extra Trees achieved cognitive status accuracy of 0.936, balanced accuracy of 0.918, macro F1 of 0.923, and ROC AUC of 0.978. MMSE, GDS, physical activity, and glucose level were influential cognitive predictors.

**Conclusion:**

The two pipelines produced strong technical discrimination and interpretable outputs, but we evaluated them on independent, unlinked datasets. The physical cohort was not confirmed as geriatric; participant- or session-level groupings were unavailable, and the provenance of the cognitive data and the target construction remain uncertain. The findings therefore support a retrospective proof of concept for separate clinician-supervised decision-support components, not for patient-level multimodal fusion, diagnosis, treatment effectiveness, recovery, or autonomous care.

## 1 Introduction

Rehabilitation aims to maximize function and minimize disability for individuals with health problems, but is not universally available in settings and populations (1). This is exacerbated by population aging, which often occurs alongside multimorbidity, frailty, mobility issues, and poorer cognitive function, all of which worsen the situation. Demographic change is leading to a growing number of people in need of long-term support, and it is estimated that around one third of the world’s population can benefit from rehabilitation at some point in illness or injury (2). Older adults may need repeated assessment, progression, and integration across physical, cognitive, psychological, and social domains. Face-to-face services are important, but travel burden, staffing levels, rural access, cost, and service disruptions can limit the frequency of contact needed to sustain long-term behavior change and functional recovery.

Telerehabilitation is an effective way to reach people in their homes and communities. The certainty and consistency of evidence for remotely delivered physical therapy vary by population, intervention, technology, and outcome, and evidence syntheses indicate it may yield outcomes similar to in-person physical therapy for some conditions (3). In stroke care, tele-rehabilitation can enhance continuity and access, but previous trials have varied in design and results, with no evidence of superiority across all clinical outcomes (4). AI can support these functions by converting camera, wearable, and/or questionnaire data into meaningful evaluations. Computational rehabilitation research has used joint trajectories, depth cameras, inertial sensors, and RGB videos to identify exercises, assess movement quality, count repetitions, and detect compensatory patterns (5). A systematic review of AI-supported physical rehabilitation applications identified several promising applications, yet highlighted the need to validate, report, and implement AI in physical rehabilitation, and to demonstrate utility beyond mere novelty (6). Recent studies have demonstrated that relationships among body joints can be learned to assess rehabilitation tasks using skeleton-based graph convolutional networks (7), and that rehabilitation tasks can be successfully evaluated with consumer cameras using a vision-based system (8). Research on video-based recognition and segmentation has also documented high accuracy in exercise recognition in controlled settings (9). Physical performance is not the only factor in healthy aging. Cognitive impairment can impair understanding instructions, recalling exercises, assessing risk, and complying with a home exercise program. At the same time, cognitive vulnerability can be accompanied by some factors such as mobility limitation, depression, poor sleep, chronic disease, and low physical activity. Brief instruments like the Mini-Mental State Examination (MMSE) and the Geriatric Depression Scale (GDS) (10) are commonly used to evaluate older adults, but they must be interpreted and should not be replaced by a non-validated algorithm. Machine-learning research, however, has shown that consistently collected variables can identify people for additional evaluation. Na (11) developed a model to predict cognitive impairment over 2 years among older adults in the community, with an area under the receiver operating characteristic (ROC) curve (AUC) of 0.921. Later models have included a range of sociodemographic, behavioral, disease history, laboratory, and cognitive factors, and performance depends critically on the target definition and whether baseline screening scores are included (12, 13). Accordingly, this study evaluates two independent machine learning pipelines within a conceptual telerehabilitation architecture, without claiming patient-level integration or clinical validation. The physical module classified three rehabilitation exercises and their execution quality from blurred video recordings using engineered spatiotemporal descriptors and conventional machine learning ensembles. The cognitive module analyzed demographic, lifestyle, clinical, and psychological data to classify cognitive impairment status.

The objectives of this study are:

- To audit both public datasets and establish reproducible analytic cohorts.
- To compare multiple algorithms using hold-out testing and stratified cross-validation.
- To quantify class-specific performance, uncertainty, calibration, and feature importance.
- To evaluate clinically meaningful physical subtasks, including exercise recognition and correctness assessment.
- To demonstrate a transparent conceptual decision support interface while keeping the two module outputs analytically separate.
- To compare the observed results with selected existing literature while identifying limits to direct benchmarking.

All analyses were conducted as a ‘technical assessment’ of the public information, on a retrospective basis. The study thus concentrates on reproducibility, uncertainty, and clear usage limits, rather than clinical effectiveness or integration at the patient level.

## 2 Materials and methods

This section describes the study design, datasets, data preprocessing, feature extraction, model development, evaluation criteria, and conceptual decision-support logic. The study includes 2 separate analytical pipelines: one using rehabilitation video data and the other using demographic, clinical, lifestyle, psychological, and cognitive-assessment data. The pipelines are trained and evaluated in isolation because they lack shared participants or a linking key.

### 2.1 Study design and reporting principles

This retrospective study uses secondary data and two independent machine learning pipelines. Patient-level fusion was not done, nor was it tested, because no individual was found in both datasets and no linkage key was available. The workflow included recursive file discovery, data quality auditing, duplicate removal, a stratified train-test split, cross-validation on the training data, final evaluation on the held-out test data, and the export of tables, figures, feature matrices, fitted pipelines, and a machine-readable manifest. Prespecification and reporting of outcomes, predictors, validation procedures, intended use, and limitations were reported transparently.

Analyses were performed in Python with a random seed of 42. Only variables and labels in the public datasets were utilized. Within the training data, candidate algorithms were selected via stratified five-fold cross-validation, with the macro F1 measure as the primary selection criterion, given the multiclass or class-imbalanced nature of the tasks. The 20% holdout sets were reserved for final performance evaluation after model selection. Complementary measures included accuracy, balanced accuracy, class-specific precision and recall, Matthews correlation coefficient (MCC), Cohen’s kappa, log loss, AUC, average precision, calibration, and inference time.

### 2.2 Physical rehabilitation video dataset and quality control

Physical module: WLU Rehabilitation Posture dataset (14) was used. The downloaded folder had a “blurred-video” branch, with six folder-defined classes: Arm Raise Correct, Arm Raise Incorrect, Knee Extension Correct, Knee Extension Incorrect, Sit to Stand Correct, and Sit to Stand Incorrect. We used these directory names to create labels. The public package contained 339 video files. We scanned every file using OpenCV to evaluate its size, codec, spatial resolution, duration, frame rate, and number of frames. MD5 hashing found only exact duplicates. Forty-eight files belonged to exact duplicate groups. Removing 24 redundant copies reduced the collection from 339 files to 315 unique, readable, labeled videos.

The final physical cohort included 106 arm-raise videos, 104 knee-extension videos, and 105 sit-to-stand videos. Composite class sizes ranged from 42 to 62 videos. The median video duration was 4.36 s (IQR 3.35–5.40 s), the median frame rate was 29 frames/s, and the total raw storage was 837.08 MB. After quality control, no unreadable or unknown-label videos remained. The dataset license acquired in the downloaded metadata was MIT. Ages of participants, clinical diagnoses, levels of impairment, distance between participants and the camera, and independent therapist ratings were not included in the analytic export; thus, the videos were not a clinically characterized geriatric cohort but rather a technical rehabilitation-exercise dataset. A summary of the physical audit is included in Table 1, and the class-level profile is reported in Table 2.

**Table 1:** Physical dataset characteristics and analytic cohort.

Characteristic	Value	Operational interpretation
Raw video files	339	All files discovered recursively
Readable, labeled, unique videos	315	One representative retained per exact-duplicate set
Files participating in duplicate sets	48	Equivalent to 24 redundant copies when duplicate pairs were collapsed
Exercises	3	Arm raise; knee extension; sit-to-stand
Execution categories	2	Correct; incorrect
Composite classes	6	Exercise x execution quality
Median duration	4.36 s	IQR 3.35–5.40 s
Median frame rate	29 frames/s	Video metadata audit
Total raw size	837.08 MB	Before analytic feature extraction

The physical dataset did not provide sufficiently detailed participant-level age or clinical metadata to confirm that it was a geriatric sample.

**Table 2:** Physical video class profile.

Composite class	*N*	Mean duration (s)	Duration SD (s)	Mean file size (MB)
Arm Raise | Correct	51	4.88	1.20	1.96
Arm Raise | Incorrect	55	3.84	1.28	1.58
Knee Extension | Correct	42	4.06	1.64	3.41
Knee Extension | Incorrect	62	3.97	1.00	1.69
Sit to Stand | Correct	57	6.30	1.59	3.42
Sit to Stand | Incorrect	48	4.19	1.60	2.12

Counts sum to 315 after quality control and duplicate handling.

### 2.3 Physical feature engineering

The physical module relied on privacy-preserving handcrafted descriptors rather than identity-recognition features to limit reliance on identifiable appearance and to avoid storing face embeddings. The 24 frames at approximately equal time points in each video were sampled and reduced to 128 × 128 pixels. Grayscale histograms, HSV histograms, brightness, contrast, sharpness, and edge density summarized appearance descriptors. Foreground-area statistics, centroid displacement, bounding-box aspect ratio, and Hu moments served as silhouette and shape descriptors. Gradient orientation was a feature indicating local posture and edge structure.

The dense optical flow with polynomial expansion (15) was used to quantify the temporal movement. The scale and direction of movement were derived from frame-to-frame flow magnitude, horizontal and vertical components, angle distributions, motion area, and frame-difference statistics. Rhythm descriptors were summarized from autocorrelation peaks, dominant frequency, spectral energy, and approximate counts of movement peaks. The metadata for videos were: duration, frame rate, width, height, and file size. Each video had 182 predictors in the total feature matrix. To enable ablation analysis and interpretability, the families were kept intact.

### 2.4 Cognitive health dataset and preprocessing

The Community Elderly Cognitive Health dataset (16) was used for the cognitive module. The analyzed file had 5,000 records and 16 columns, with no missing field values and no duplicate records. The outcome was cognitive_impairment_status (0 = no cognitive impairment, 1 = cognitive impairment). Participant ID was removed. Fourteen predictors were left: age, gender, education level, region, marital status, chronic diseases, glucose level, body mass index, MMSE score, GDS score, sleep-quality score, physical-activity score, smoking status, and alcohol use. The model included nine numeric and five categorical predictors. No sensitivity analysis excluding MMSE and GDS was performed in the present study. Consequently, the analysis does not demonstrate incremental predictive value beyond these target-proximal variables and should not be interpreted as independent screening, early detection, diagnosis, or prediction of future cognitive impairment. The cognitive-impairment outcome comprised 3,500 records in the negative class (class 0; 70%) and 1,500 records in the positive class (class 1; 30%). The median imputation technique was used for numeric variables and the most frequent imputation technique, or one-hot encoding, for categorical variables. This version had no missing values, but the imputation steps were still included in the fitted pipeline to preserve the system’s robustness when deployed in the field. The source page for the expanded 5,000-record file does not document recruitment, clinical adjudication, or the data-generation protocol. Thus, the clinical, synthetic, or augmented provenance cannot be analyzed independently and is regarded as a significant drawback.

MMSE and GDS were included because they were present in the source dataset, but given their proximity to cognitive impairment, they risk incorporation bias. Because the source does not document how cognitive_impairment_status was generated, the observed performance is interpreted as technical concurrent status classification rather than evidence of independent screening or future risk prediction. A model excluding MMSE and GDS would be required to quantify the incremental value of the remaining predictors. The cohort and preprocessing decisions are summarized in Table 3.

**Table 3:** Cognitive dataset characteristics and preprocessing decisions.

Characteristic	Observed value	Methodological implication
Records analyzed	5,000	Expanded CSV file selected
Target distribution	3,500 negative; 1,500 positive	70:30 class ratio
Retained predictors	14	9 numeric; 5 categorical
Missing values	0	Imputation retained in pipeline for deployment robustness
Duplicate rows	0	No row-level duplicates detected
Removed identifier	participant_id	Unique identifier excluded from modeling
Target-proximal cognitive variables	MMSE; GDS	No sensitivity analysis excluding MMSE/GDS was performed; incremental predictive value beyond these variables was not established
Source provenance	Kaggle repository	Clinical/synthetic status not independently verified

High apparent model performance must be interpreted in light of uncertain data provenance and the inclusion of MMSE/GDS.

### 2.5 Model development

In the physical 6-class outcome, the performance of five supervised classifiers – multinomial logistic regression, radial basis function support vector machine, random forest, Extra Trees, and histogram gradient boosting – was compared. Random forest and Extra Trees are ensemble methods based on decorrelated decision trees (17, 18), while histogram gradient boosting iteratively fits the residual structure based on a gradient-based objective, similar to existing boosting techniques (19). No scaling was done unless necessary, especially for logistic regression and support vector machine. Discrimination, calibration, uncertainty, and referral analyses were performed to generate probability estimates.

Logistic regression, random forest, Extra Trees, and gradient boosting were tested as models in a single preprocessing and model pipeline for the cognitive binary outcome. Appropriate hyperparameter values were selected to avoid overfitting on a public dataset and ensure computational repeatability. An 80:20 stratified split was applied to both datasets. Candidate algorithms and hyperparameters were compared only within the 80% training partition using five-fold stratified cross-validation and macro F1 as the primary selection criterion. After the model choice was fixed, the untouched 20% holdout partition was used for final evaluation, with balanced accuracy, calibration, agreement measures, and inference time reported as complementary criteria. Table 4 presents the full development and validation protocol. All analyses used a fixed random seed of 42. For both modules, a stratified 80:20 development–holdout split was used. Candidate models were compared by stratified five-fold cross-validation within the 80% development partition using macro-F1 as the primary selection criterion, after which the selected model was evaluated once on the untouched 20% holdout partition. For the physical pipeline, each video was represented by 182 handcrafted predictors extracted from 24 uniformly sampled frames resized to 128 × 128 pixels. Standardization was applied to Logistic Regression and RBF-SVM, whereas the tree-based models were fitted to the unscaled handcrafted features. For the cognitive pipeline, the 14 retained predictors comprised nine numeric and five categorical variables. Numeric variables were processed using median imputation, while categorical variables used most-frequent imputation followed by one-hot encoding; all preprocessing was fitted within the training pipeline. For the physical module, the selected Random Forest classifier used 600 trees (n_estimators = 600), square-root feature sampling at each split (max_features = ‘sqrt’), a minimum of two samples per leaf (min_samples_leaf = 2), balanced-subsample class weighting (class_weight = ‘balanced_subsample’), and a random seed of 42. For the cognitive module, the selected Extra Trees classifier used 500 trees (n_estimators = 500), a minimum of two samples per leaf (min_samples_leaf = 2), balanced class weighting (class_weight = ‘balanced’), and a random seed of 42. Parallel tree fitting was enabled for both models (n_jobs = −1); estimator parameters not explicitly specified retained their scikit-learn defaults.

**Table 4:** Model development and evaluation protocol.

Module	Candidate algorithms	Outcome	Validation	Primary selection metric
Physical	Logistic regression; RBF-SVM; random forest; Extra Trees; histogram gradient boosting	Six composite classes	5-fold stratified CV within the 80% training set; final evaluation on untouched 20% holdout	Training CV macro-F1
Cognitive	Logistic regression; random forest; Extra Trees; gradient boosting	Binary impairment status	5-fold stratified CV within the 80% training set; final evaluation on untouched 20% holdout	Training CV macro-F1
Physical subtasks	Locked six-class Random Forest predictions collapsed *post hoc*; no separate retraining	Exercise identity; correct/incorrect	Same 63 hold-out records	Accuracy, balanced accuracy, macro-F1, MCC
Decision support	Prespecified rules from probabilities/confidence	Action/referral category	Illustrative only	Transparent thresholds; no clinical outcome claim

CV, cross-validation; MCC, Matthews correlation coefficient; RBF-SVM, radial-basis-function support vector machine.

### 2.6 Performance measures, confidence intervals, and explainability

Accuracy uncertainty was estimated by nonparametric bootstrap resampling of the held-out observations with replacement using 500 bootstrap repetitions. Accuracy was recalculated for each bootstrap sample, and the 2.5th and 97.5th percentiles of the resulting bootstrap distribution were used as the 95% confidence interval. The same procedure was applied to the 63-video physical holdout and the 1,000-record cognitive holdout. Balanced accuracy was the average of the class recalls. Weighted F1 accounted for class frequency, while macro precision, macro recall, and macro-F1 were weighted equally across classes. Beyond overall classification performance, we assessed agreement using MCC and Cohen’s kappa. The probability quality was evaluated by multiclass log loss. When the positive class is less numerous, we summarize binary cognitive discrimination using ROC-AUC and average precision. The precision, recall, F1, and support values are reported per class for both modules.

To measure the loss in prediction accuracy, we randomly permuted one feature at a time and assessed the impact using permutation importance. This model-agnostic alternative approach was favoured for interpreting manuscripts and complemented tree specificity. Permutation importance was used as a model-agnostic feature dependence measure within a broader interpretable machine learning framework (20). The reporting decisions also followed the main principles of TRIPOD and prediction model transparency (21). The physical ablation analysis was conducted as a separate refitting experiment rather than by masking features only at inference. Five predefined feature configurations were evaluated: video metadata (5 features), appearance (60 features), shape-gradient (70 features), motion-temporal (47 features), and the complete set of 182 features. Each configuration was refitted and evaluated using the same predefined holdout records and random seed 42. The ablation analysis was separate from the locked primary-model evaluation; therefore, the all-feature ablation result was not treated as the primary Random Forest estimate.

### 2.7 Physical subtasks and conceptual decision-support logic

The six physical labels were collapsed into two secondary analytic tasks. The exercise identity task ignored correctness and classified arm raise, knee extension, or sit-to-stand, whereas the correctness task ignored exercise identity and classified the dataset-defined execution label as correct or incorrect. This decomposition separates activity recognition from the more difficult execution-label classification. The physical subtask analyses did not involve retraining separate models. Instead, the held-out predictions of the locked six-class Random Forest were collapsed after inference. For exercise recognition, the correct/incorrect component was removed from both the true and predicted six-class labels, leaving three exercise categories: arm raise, knee extension, and sit-to-stand. For execution-quality assessment, exercise identity was removed, leaving the binary correct/incorrect categories. Accuracy, balanced accuracy, macro-F1, and MCC were then recalculated from these collapsed labels on the same 63 held-out videos. For illustration only, predicted class probabilities and confidence can be mapped to workflow prompts such as accept, re-record, or clinician review. Low-confidence or dataset-defined incorrect outputs should prompt re-recording or professional review rather than autonomous corrective, safety, or prescription decisions.

It is retained only for conceptual interface illustration purposes and is not included in the empirical model performance evaluation. The two modules were not integrated on a patient-by-patient basis. An overall physical-cognitive score was not computed or validated, and the physical and cognitive outputs are treated separately in all empirical analyses. The information in the workflow prompts is illustrative, not a learning or clinically proven treatment pathway. The scenarios illustrate how separate module outputs could be presented in a future interface, but do not suggest empirical fusion, patient-level risk estimation, or clinical validation. The two-stream architecture and the deliberately small decision-support junction are shown in Figure 1.

**Figure 1:**
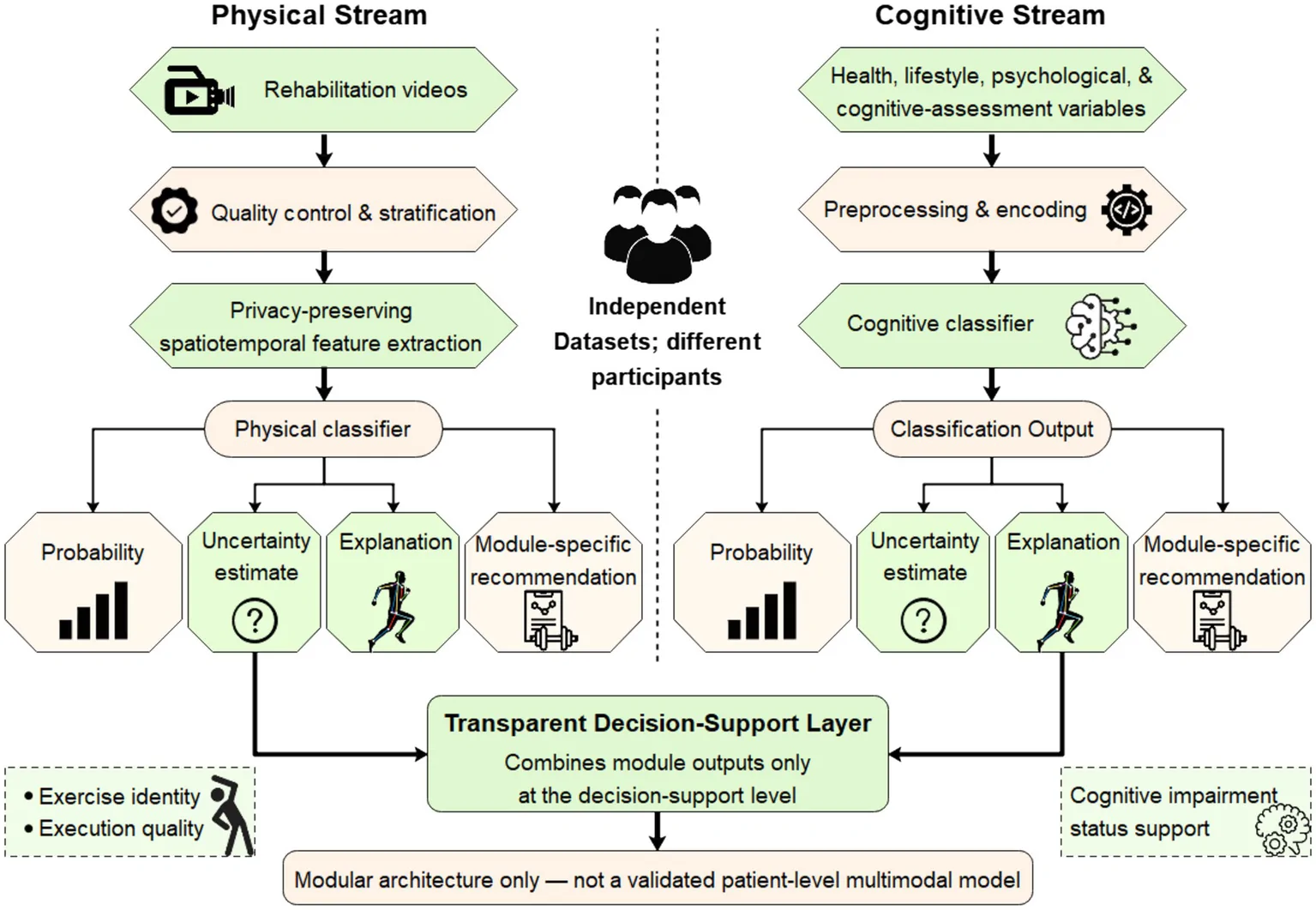
Dual-module architecture. The physical stream analyses rehabilitation videos, and the cognitive stream analyses health, lifestyle, psychological, and cognitive-assessment variables.

### 2.8 Ethical and safety considerations

The study used existing secondary data and did not recruit, contact, intervene with, or reidentify any new people. Physical videos were provided in a blurred version, and there was no face recognition, identity matching, or biometric identification. In accordance with the authors’ applicable institutional policy, no new ethics approval or additional informed consent was required for the secondary analysis of these publicly released de-identified datasets. It was not possible to reuse all the data, as reuse was restricted to publicly released materials and the stated reuse conditions.

The purpose is to support the clinician, not to diagnose or treat. Clinicians should not use model output alone to drive an exercise prescription or to make safety decisions when pain, instability, cardiopulmonary symptoms, and/or other concerns exist. Pre-deployment usability testing and accessibility adaptation, cybersecurity review, medical device regulatory assessment, and clinical validation in suitably characterized older adult populations are necessary before deployment.

## 3 Results

### 3.1 Dataset audit and descriptive findings

Physical Check found 339 videos and 0 unreadable files. They then merged exact duplicates; 315 videos remained across 6 classes. The class distribution was moderately non-normal, with Knee Extension Incorrect as the largest class (*n* = 62) and Knee Extension Correct as the smallest (*n* = 42). The mean duration of sit-to-stand correct videos was 6.30 s, whereas the arm raise incorrect videos had the shortest duration at 3.84 s. Descriptive statistics of the physical rehabilitation video dataset are shown in Figure 2. Figure 2a depicts the results for correct versus incorrect recordings for the three exercises. The highest number of incorrect videos was recorded with knees extended, while the highest number of correct videos was recorded during sit-to-stand. The distributions of the video duration for each study are shown in Figure 2b; sit-to-stand had the widest distribution and longest video length, while arm-raise and knee-extension video duration distributions were more concentrated at shorter durations. Summary of the most common spatial resolution, as shown in Figure 2c, revealed that 1,280 × 720 was the most prevalent video resolution, followed by a few with lower and higher resolutions. The duration of the recordings is generally related to the size of the recording file, as shown in Figure 2d; however, some outliers are quite large, indicating that recording settings and compression efficiency may have varied.

**Figure 2:**
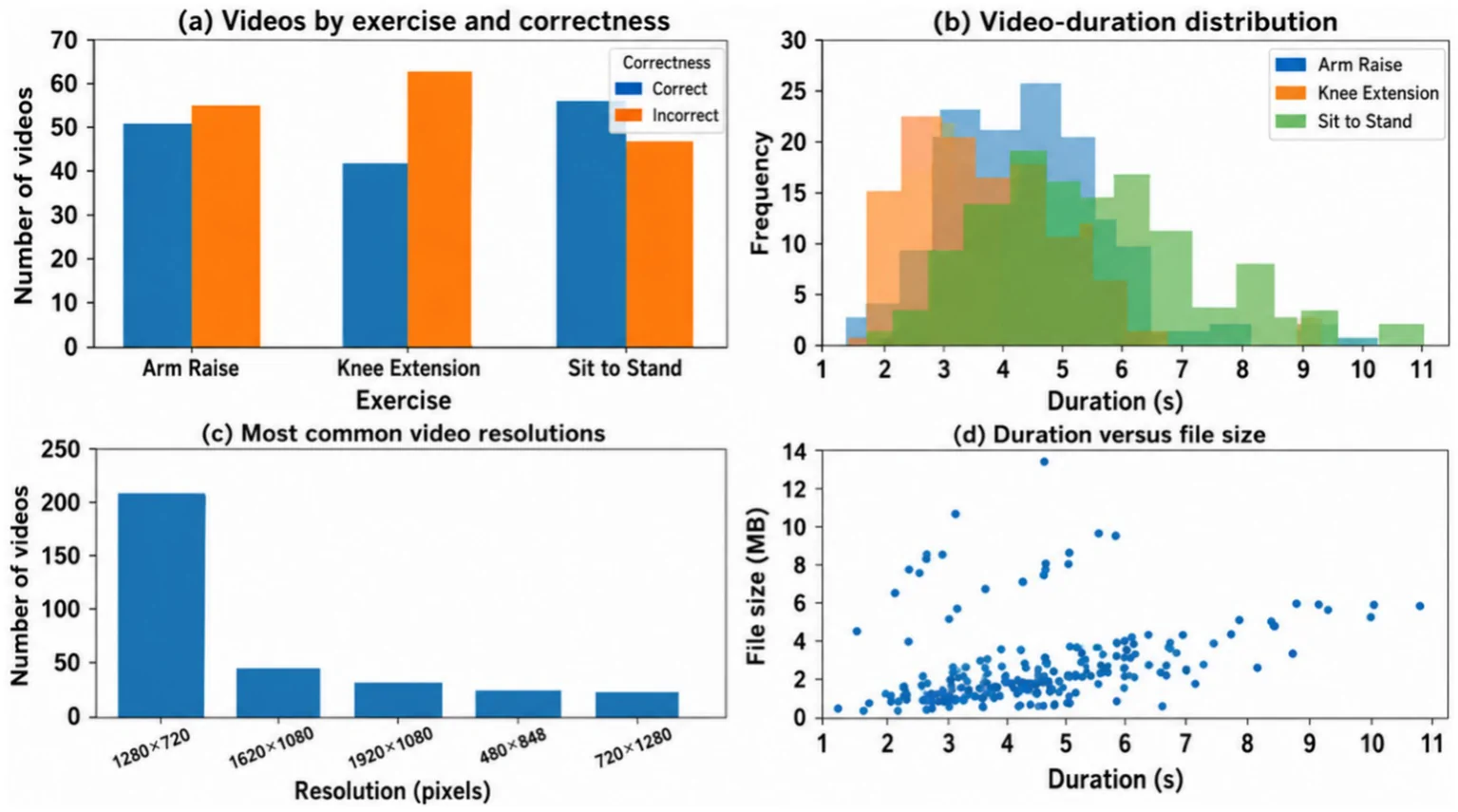
Physical dataset exploration. **(a)** Video counts by exercise and execution correctness. **(b)** Video-duration distributions by exercise. **(c)** Most common spatial resolutions. **(d)** Relationship between video duration and file size. The panels show within-exercise correctness imbalance, a broader duration distribution for sit-to-stand, heterogeneous acquisition resolutions, and the expected positive association between recording duration and storage size.

The cognitive file contained 5,000 complete records. Class 0 comprised 70% of records, and class 1 comprised 30%. The 14 predictors retained were demographic, clinical, lifestyle, and psychological variables with no recorded missingness. Figure 3 summarizes the main characteristics of the cognitive-health dataset. The binary target distribution is shown in Figure 3a, where there are 3,500 records in class 0 (cognitively normal) and 1,500 records in class 1 (cognitively impaired), giving a class ratio of 70:30. The profile of the missing values from the retained predictors is shown in Figure 3b and indicates that there are no missing values in the variables analyzed. Figure 3c shows the raw variation of numeric predictors: glucose level had the greatest standard deviation, followed by age, MMSE score, body mass index, and GDS score. These values indicate the scales of measurement and should not be interpreted as predictive importance. Figure 3d shows the predictor-type composition (64.3% numeric variables and 35.7% categorical variables).

**Figure 3:**
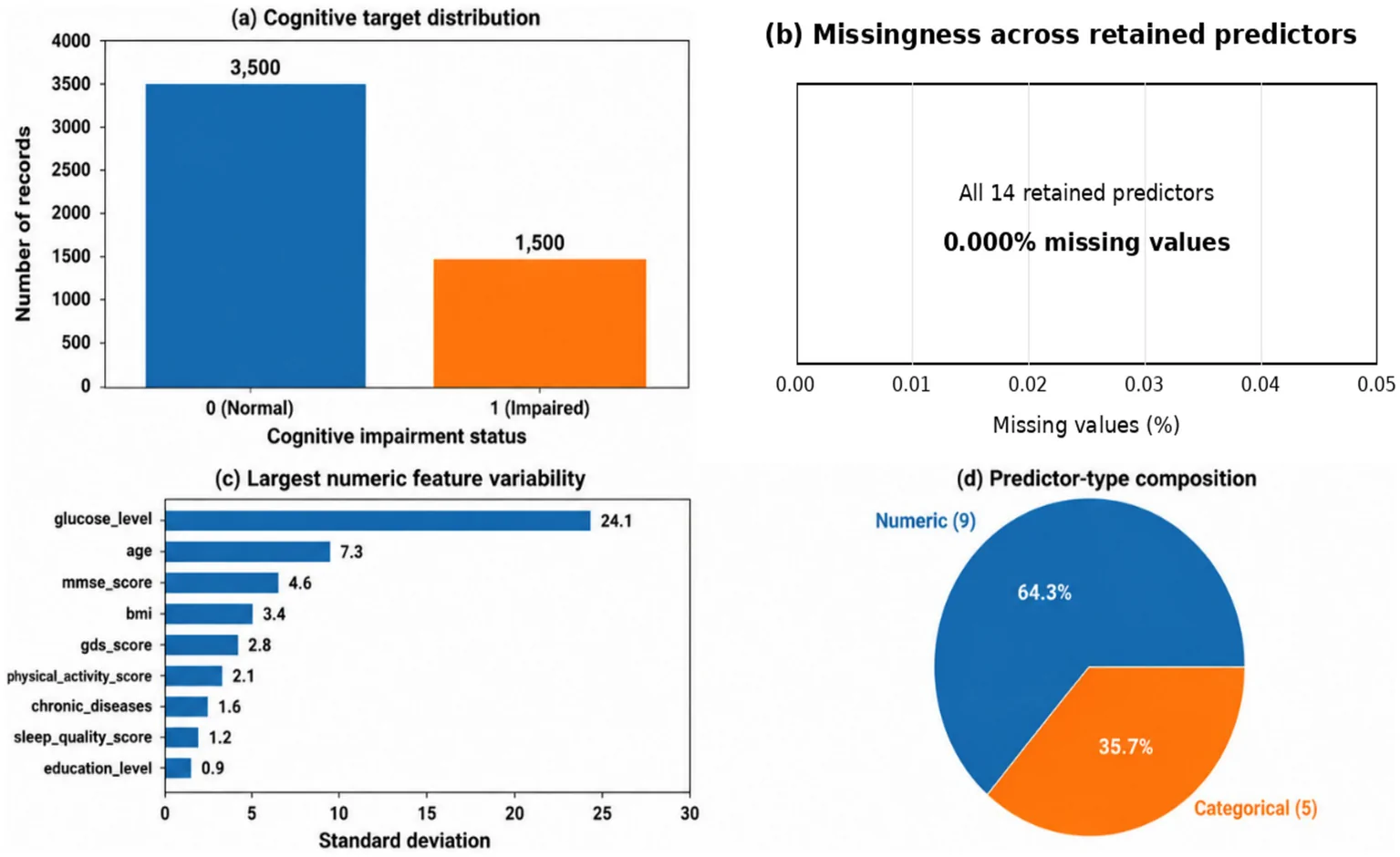
Cognitive dataset exploration. **(a)** Binary target distribution. **(b)** Missingness percentages for retained predictors; all were zero. **(c)** Numeric predictors with the largest raw standard deviations. **(d)** Predictor-type composition, comprising nine numeric and five categorical variables. These descriptive panels characterize data structure and do not indicate predictive importance.

### 3.2 Physical model performance

Random forest, selected from the training data by cross-validation macro F1, achieved held-out six-class accuracy of 0.857 (95% bootstrap CI 0.778-0.937), balanced accuracy of 0.846, macro precision of 0.859, macro recall of 0.846, macro-F1 of 0.849, weighted F1 of 0.860, MCC of 0.829, and Cohen’s kappa of 0.828. For transparency, we report holdout metrics for the remaining candidate classifiers only as post-selection descriptive comparisons; we did not use them for model selection, tuning, or revision of the locked random forest model. Specific figures are given in Table 5.

**Table 5:** Held-out performance of physical six-class classifiers.

Model	Accuracy	95% CI	Balanced accuracy	Macro-F1	MCC	Kappa	Inference ms/video
Random Forest	0.857	0.778-0.937	0.846	0.849	0.829	0.828	2.12
Hist. Gradient Boosting	0.841	0.746-0.921	0.840	0.836	0.813	0.809	0.46
Extra Trees	0.825	0.730-0.921	0.822	0.821	0.792	0.790	2.60
Logistic Regression	0.746	0.635-0.841	0.741	0.737	0.697	0.694	0.07
RBF-SVM	0.730	0.619-0.841	0.730	0.730	0.682	0.676	0.06

Random forest was selected before holdout inspection using training-data cross-validation macro-F1. Holdout metrics for nonselected algorithms are post-selection descriptive comparisons only and were not used for model choice or hyperparameter tuning. Confidence intervals are bootstrap intervals for holdout accuracy.

These are video-level performance estimates. Because participant and session identifiers were unavailable, we could not group the split by individual or recording session, and it should not be interpreted as evidence of subject-independent generalization.

### 3.3 Physical class-specific errors and discrimination

There was a wide range of performance within each class. Precision, recall, and F1 for sit-to-stand correct were 1.000 for 12 test videos, while precision, recall, and F1 for sit-to-stand incorrect were 0.947. F1 for Arm Raise Correct and Arm Raise Incorrect were 0.889 and 0.917, respectively. Quality of knee extension was more challenging: Knee Extension Correct had precision 0.556, recall 0.625, and F1 0.588, while Knee Extension Incorrect had F1 0.750 (Table 6).

**Table 6:** Class-specific performance of the selected physical model.

Composite class	Precision	Recall	F1	Test *n*
Arm Raise | Correct	1.000	0.800	0.889	10
Arm Raise | Incorrect	0.846	1.000	0.917	11
Knee Extension | Correct	0.556	0.625	0.588	8
Knee Extension | Incorrect	0.750	0.750	0.750	12
Sit to Stand | Correct	1.000	1.000	1.000	12
Sit to Stand | Incorrect	1.000	0.900	0.947	10

The weakest class was Knee Extension Correct, while both sit-to-stand classes showed strong test performance.

The confusion matrices for the three best physical-classification models are compared row-wise, in the sense that the rows are normalized, as shown in Figure 4. The Histogram Gradient Boosting and Extra Trees panels are post-selection descriptive diagnostics and did not influence model choice. For the locked Random Forest model, with the majority of arm-raise and sit-to-stand recordings being correctly classified (Figure 4a). The main errors were between correct knee-extension and incorrect knee-extension, with 37.5% of correct videos being counted as incorrect and 25.0% of incorrect videos being counted as correct. The error pattern for Histogram Gradient Boosting is also similar (Figure 4b), but slightly weaker during sit-to-stand and knee extensions. The results of Extra Trees are shown in Figure 4c, which classified both sit-to-stand classes well but was less successful at assessing arm-position correctness and knee-extension quality.

**Figure 4:**
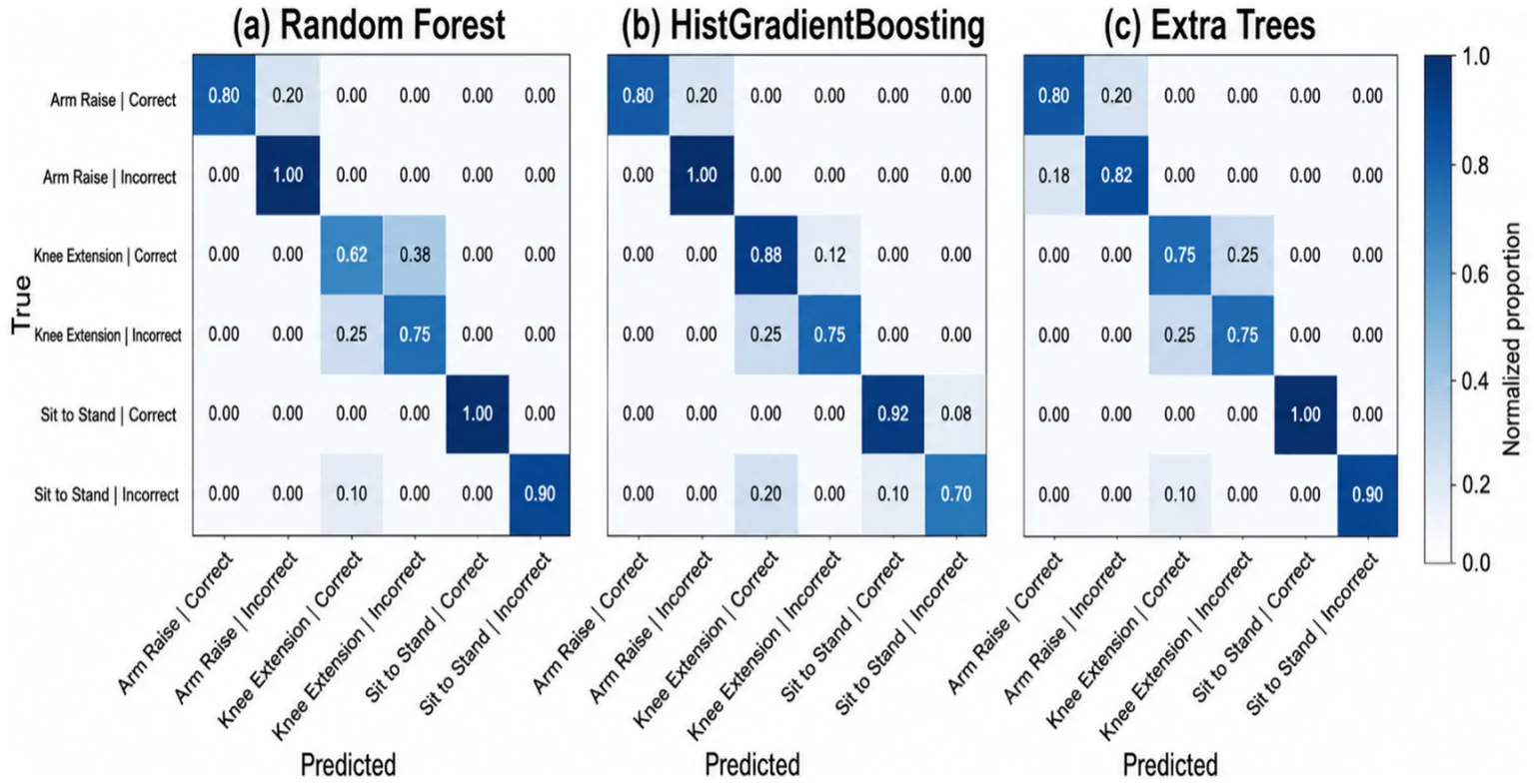
Normalized physical confusion matrices. **(a)** Random forest. **(b)** Histogram gradient boosting. **(c)** Extra Trees. Rows indicate true classes and columns indicate predicted classes. The dominant residual confusion occurs between correct and incorrect knee extension.

### 3.4 Physical feature families, uncertainty, and physical subtasks

Both the permutation and ablation analyses showed that structural image information was particularly helpful. In the ablation protocol, the shape-gradient features achieved an accuracy of 0.841, a balanced accuracy of 0.834, and a macro-F1 of 0.835 with 70 features. The appearance-only model achieved an accuracy of 0.746, whereas the video metadata-only and motion temporal-only models each achieved 0.524. The all-feature refit in the same ablation protocol achieved an accuracy of 0.810, as summarized in Table 7.

**Table 7:** Physical feature-family ablation results.

Feature family	Features	Accuracy	Balanced accuracy	Macro-F1	MCC
Shape-gradient	70	0.841	0.833	0.835	0.810
All features	182	0.810	0.806	0.804	0.773
Appearance	60	0.746	0.736	0.736	0.695
Video metadata	5	0.524	0.520	0.512	0.428
Motion-temporal	47	0.524	0.519	0.487	0.432

Ablation values arise from a dedicated refitting protocol and are not directly interchangeable with the final model’s Table 5 hold-out estimate.

Figure 5 summarizes the uncertainty, calibration, selective prediction, and error characteristics of the model selected as a physical classification model. Figure 5a shows predictive-confidence distributions for correct and incorrect classifications. Correct predictions extended across a broader confidence range, whereas errors were concentrated mainly at lower to moderate confidence, particularly around 0.4 to 0.6. To illustrate this, the relationship between mean predicted confidence and accuracy is shown in Figure 5b as the confidence-calibration graph. Figure 5b shows the relationship between predictive confidence and observed accuracy. Observed accuracy generally exceeded the corresponding mean predicted confidence, indicating that the model tended to be underconfident rather than overconfident. Figure 5c shows the uncertainty-based referral trade-off. As the confidence threshold increased, the proportion of retained cases decreased, whereas selective accuracy among the retained predictions improved and approached 1.00. This pattern suggests a confidence-based strategy for flagging low-confidence cases for re-recording or human review; however, we did not evaluate its clinical utility. Figure 5d shows the most frequent error transitions.

**Figure 5:**
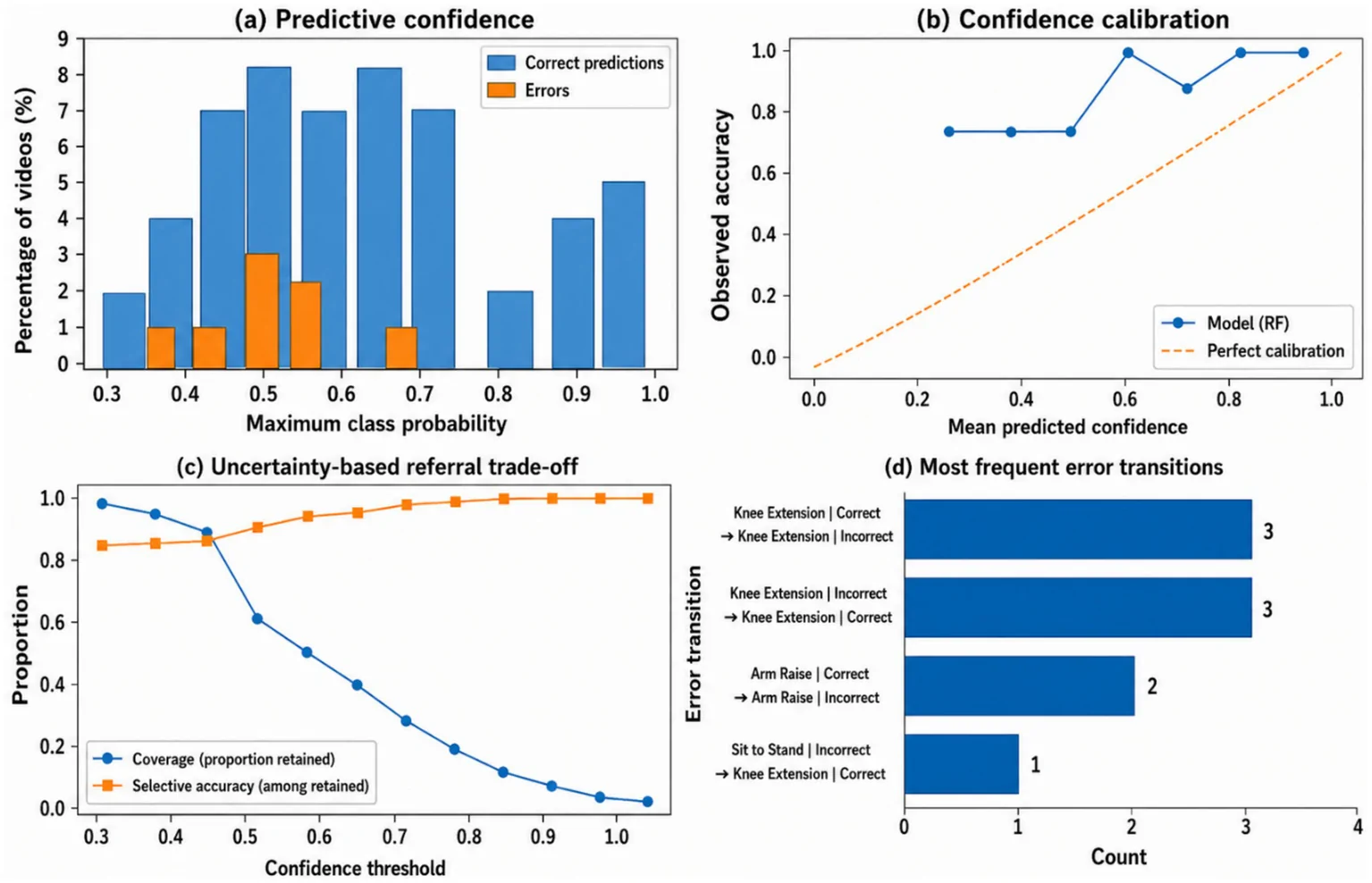
Physical uncertainty and error analysis. **(a)** Predictive-confidence distribution for correct predictions and errors. **(b)** Calibration of mean confidence against observed accuracy. **(c)** Referral-threshold trade-off between retained coverage and selective accuracy. **(d)** Most frequent true-to-predicted error transitions.

Failing labels helped to identify model successes. The accuracy of the identified exercise was 0.984, while balanced accuracy was 0.985; macro-F1 was 0.984, and MCC was 0.977. With correctness-only assessment, accuracy, balanced accuracy, macro-F1, and MCC were 0.857, 0.856, 0.857, and 0.714, respectively. The 6-class composite task achieved an accuracy of 0.857 and a macro-F1 of 0.849. The exact metrics for each subtask are reported in Table 8.

**Table 8:** Physical subtask performance.

Task	Accuracy	Balanced accuracy	Macro-F1	MCC
Exercise recognition	0.984	0.985	0.984	0.977
Correct/incorrect execution	0.857	0.856	0.857	0.714
Six-class composite assessment	0.857	0.846	0.849	0.829

Exercise-recognition and correctness metrics were calculated by post hoc collapsing of the true and predicted labels from the locked six-class Random Forest on the same 63 held-out videos. No separate subtask models were retrained. Exercise recognition ignores execution quality, correctness assessment ignores exercise identity, and the composite assessment retains all six classes.

### 3.5 Cognitive model performance and explanations

Extra Trees was the selected classifier for the concurrent cognitive status task. On the 1,000-record hold-out set, it achieved accuracy 0.936 (95% bootstrap CI 0.920-0.950), balanced accuracy 0.918, macro precision 0.928, macro recall 0.918, macro-F1 0.923, weighted F1 0.936, MCC 0.846, kappa 0.846, log loss 0.237, ROC-AUC 0.978, and average precision 0.957. For transparency, holdout metrics for the remaining classifiers are reported only as post-selection descriptive comparisons; they did not influence training-data model selection or hyperparameter tuning. Table 9 summarizes the complete holdout metrics for all candidate classifiers.

**Table 9:** Held-out performance of cognitive classifiers.

Model	Accuracy	95% CI	Balanced accuracy	Macro-F1	MCC	ROC-AUC	Average precision
Extra trees	0.936	0.920-0.950	0.918	0.923	0.846	0.978	0.957
Random forest	0.931	0.916-0.945	0.910	0.916	0.834	0.978	0.959
Gradient boosting	0.928	0.914-0.943	0.907	0.913	0.827	0.978	0.959
Logistic regression	0.924	0.907-0.939	0.921	0.911	0.824	0.979	0.959

Extra Trees was selected before holdout inspection using training-data cross-validation macro-F1. Holdout metrics for the remaining classifiers are post-selection descriptive comparisons only and were not used for model selection or tuning. All models in this table use the 14-predictor specification, including MMSE and GDS; therefore, the results quantify within-dataset classification rather than independent screening performance.

Given the uncertain provenance and undocumented construction of cognitive_impairment_status, together with the prominence of MMSE and GDS, these values quantify technical discrimination only within this dataset. They are not interpreted as independent cognitive risk prediction or clinical validation. The 14-predictor specification includes MMSE and GDS, so read the discrimination results as an augmented within-dataset classification analysis rather than a target-independent screening estimate.

The discrimination performance of the model adopted for classifying cognitive categories is shown in Figure 6. The confusion matrix is presented in absolute numbers in Figure 6a. The model correctly classified 674 records from class 0 and 262 records from class 1; and 26 class-0 records were classified as class 1 and 38 class-1 records were classified as class 0. The confusion matrix is shown in Figure 6b, which is the row-normalized version of the original confusion matrix. The model had a recall of 0.96 for class 0 and 0.87 for class 1, indicating that it is better at recognizing the non-impaired group and has a relatively high false-negative rate for the impaired group. The 1-vs-rest receiver operating characteristic (ROC) curves are shown in Figure 6c. The area under the curve for both classes was around 0.98, indicating that both classes were discriminated well at all classification thresholds. The precision–recall curves are plotted on the right in Figure 6d. The average precision for class 0 and class 1 was around 0.99 and 0.96, respectively.

**Figure 6:**
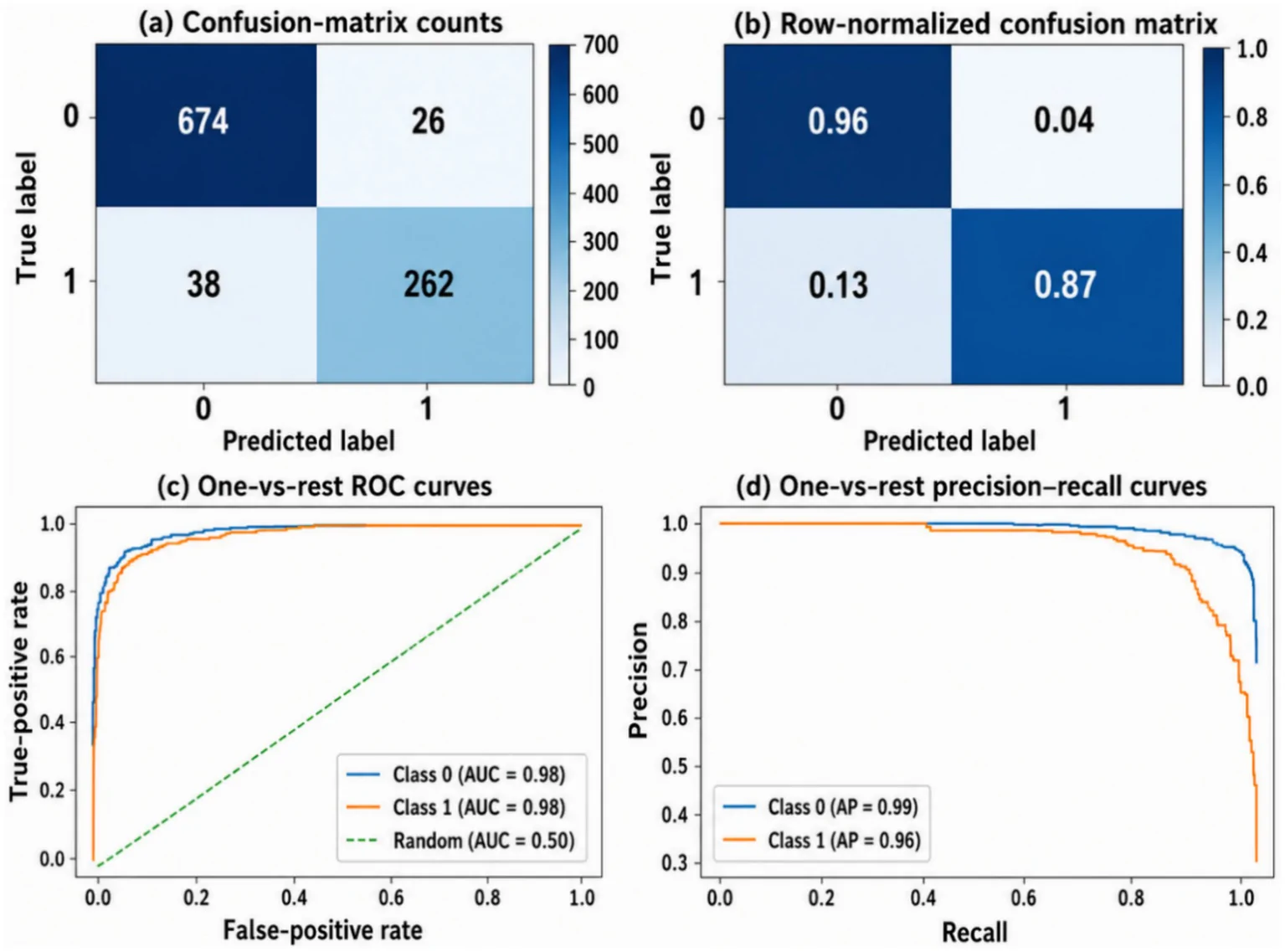
Cognitive discrimination analysis. **(a)** Confusion matrix in counts. **(b)** Row-normalized confusion matrix. **(c)** Receiver operating characteristic curve. **(d)** Precision-recall curve. The positive class had lower recall than the negative class despite high overall discrimination.

Because this 14-predictor model includes MMSE and GDS and the target-generation rule is undocumented, the figure is interpreted only as concurrent within-dataset classification. No sensitivity analysis excluding MMSE and GDS was performed; therefore, the reported discrimination does not demonstrate incremental predictive value beyond these target-proximal variables and should not be interpreted as independent screening, early detection, risk prediction, or diagnostic utility.

### 3.6 Conceptual decision-level personalization scenarios

The five scenarios were constructed solely to illustrate how separate module outputs might be displayed in a future interface; they are not paired patient observations or empirical integration results. No numerical physical-cognitive combination is used. The examples retain separate module outputs and demonstrate only qualitative interface prompts such as routine monitoring, re-recording, or clinician review. As explicitly stated in Table 10, none of the rows are paired patient evidence; rather, they are framework illustrations.

**Table 10:** Illustrative decision-level personalization scenarios.

Scenario	Physical output	Cognitive output	Combined score	Suggested action
1	Correct/high conf.	Lower concern	Not used	Routine monitoring
2	Correct/low conf.	Lower concern	Not used	Re-record or reassess
3	Incorrect label	Lower concern	Not used	Clinician review before progression
4	Correct/high conf.	Higher concern	Not used	Cognitive review before progression
5	Incorrect label	Higher concern	Not used	Clinician review; no autonomous action

All scenarios are unvalidated interface illustrations constructed from independent datasets; no combined numerical score is computed, and none of the rows represent paired patient evidence.

No inference in this study depends on these conceptual interface scenarios, and no patient-level combined score is estimated.

### 3.7 Comparison with existing literature

Selected physical-rehabilitation and cognitive-screening studies are included with the results in Table 11. It’s a comparison, not a ranking. The exercise sets, sensors, labels, participants, and validation design of physical studies vary. The result of this exercise is 98.4%, which is close to the result of repetitive activity recognition that was obtained by (9) with 99.1% accuracy, but the tasks and the dataset are different. The accuracy of 85.7% for exercise quality is less than the 90.6% (10-fold) estimate reported by (8) but better than their leave-one-subject-out estimate of 83.8%. These differences should not be interpreted as head-to-head performance differences. Instead, they illustrate how validation design, particularly subject-independent testing, can materially affect reported accuracy and reinforce the point that the present video-level split cannot establish subject-independent generalization.

**Table 11:** Contextual comparison with selected existing literature.

Study	Dataset/population	Task	Validation	Reported result
Current physical module	315 unique WLU videos; 3 exercises	Six-class exercise x quality	Training CV selection + final 20% holdout	Accuracy 85.71%; macro-F1 84.85%
Na (11)	3,424 community older adults	Two-year incident cognitive impairment	Longitudinal test	AUC 0.921; sensitivity 0.967; specificity 0.825
Wang et al. (13)	Older adults in Northwest China	Cognitive-impairment risk	Internal validation	AUC 0.816; accuracy 73.14%; F1 0.802
Ren et al. (12)	CLHLS; *n* = 2,688	Future cognitive decline	Internal + prospective validation	Accuracy 88.5% internal; 88.7% prospective
Song et al. (22)	NHANES; *n* = 2,208	Cognitive-impairment classification	Train/test evaluation	Accuracy 93.5%; AUC 0.981
Ye and Peng (23)	Routine EHR data; older outpatients	Adjudicated mild cognitive impairment	10-fold CV + held-out test	AUC approximately 0.85
Current cognitive module	Kaggle expanded data; *n* = 5,000	Concurrent impairment-status classification	Training CV selection + final 20% stratified record-level holdout	Accuracy 93.6%; macro-F1 92.3%; AUC 0.978

Metrics are not head-to-head because datasets, outcomes, and validation designs differ. LOSO, leave-one-subject-out; CV, cross-validation; AUC, area under the receiver operating characteristic curve.

The sensitivity of cognitive comparisons to outcome definitions is also demonstrated. Na (11) target structure differed from the concurrent target structure, and the AUC of her two-year incident model was 0.921. In a longitudinal study of a Chinese population, Ren et al. (12) reported accuracies of 88.5 and 88.7% in internal and prospective validation, respectively. NHANES cognitive tests and other variables achieved 93.5% accuracy and an AUC of 0.981, similar to the 93.6% accuracy and 0.978 AUC reported in the present study. Song et al. (22) reported an accuracy of 93.5% and an AUC of 0.981 using NHANES cognitive tests and other variables, results that are very close to the current 93.6% and 0.978. The similarity of these high discrimination estimates does not establish external validity because both settings include cognitive test variables strongly related to the target. In comparison, the 2026 EHR model (23) achieved AUCs around 0.85 using routine outpatient data and an adjudicated mild cognitive impairment outcome. This demonstrates the more difficult task of screening with less target-proximal information.

## 4 Discussion

The main objective of this study is to assess two independent, explainable machine learning pipelines that perform complementary technical tasks relevant to telerehabilitation: classifying rehabilitation exercises from video and classifying cognitive status from tabular records. Under random video-level holdout evaluation, the physical pipeline achieved 85.7% accuracy and an 84.9% macro F1 score for the six-class exercise-by-correctness task; participant-independent performance could not be established because participant and session identifiers were unavailable. The cognitive pipeline achieved 93.6% accuracy, a macro F1 of 92.3%, and an AUC of 0.978. These values are only technically separable within the datasets and do not represent a clinically tested integrated rehabilitation system. The key contribution is a transparent modular architecture that keeps evidence, uncertainty, explanations, and recommendations separate when patient-level linkage is unavailable.

The isolated feature families showed the strongest shape-gradient information. This pattern is plausible because the three exercises have very different global postures and edge structures. The motion temporal features, on their own, were much less significant, indicating that global optical flow might not be sufficient to explain the fine-grained inter-joint differences in the dataset labels. For knee extension, there is considerable confusion about what is correct and incorrect, suggesting that the current handwritten descriptors were less effective at discriminating subtle execution differences than at distinguishing between exercise identities. Moving forward, we need to assess pose landmarks, joint angles, phase-normalized trajectories, side-to-side symmetry, and therapist-defined error categories. Other relationships can be better represented using graph-based representations (7) or pose-specific rehabilitation models.

The physical outcomes also show the problems with separating recognition from exercise execution. Identifying which activity is being performed is easier: exercise-identity accuracy was 98.4%, compared with 85.7% accuracy and 0.714 MCC for the correctness-only task. Because therapists did not assign correctness labels and the folder name was used instead, and because there was no standardized clinical rubric, these predictions should be understood as dataset-derived labels, not as an assessment of the safety or completeness of the predictions or the appropriateness of the prescriptions. The model will thus prompt re-recording or clinician review if its confidence output is low; the model should not make decisions of its own, such as corrections or safety decisions. Across algorithms, the cognitive classifiers showed similarly high discrimination but were equally uncommon, as the target is not easily separable from the supplied predictors.

Permutation importance identified the variables contributing most strongly to the observed cognitive discrimination, with MMSE and GDS among the most influential predictors. The MMSE assesses global cognitive function, whereas the GDS is a screening instrument for depressive symptoms (10). Depressive symptoms may, in turn, influence cognitive performance and functional engagement. When cognitive_impairment_status is defined using MMSE thresholds or a function that includes MMSE and GDS, the model mostly learns the data-construction rule. Even if MMSE and GDS were not explicitly used to construct the label, an AUC of 0.978 indicates dataset separability rather than clinical discovery.

This issue changes how we should interpret the cognitive module. The analysis is best viewed as a proof of concept for concurrent status classification using cognitive-assessment, health, and lifestyle variables, not as an independent diagnostic, screening, or prognostic model. Because no sensitivity analysis excluding MMSE and GDS was performed, the present study does not establish incremental predictive value of the remaining variables beyond these target-proximal measures. Longitudinal studies clearly show the need to distinguish between concurrent classification and prediction. Na (11) model included baseline variables as the predictors of impairment after 2 years, and Ren et al., (12) added a prospective validation sample. These designs offer more credibility in risk forecasting, even if they do not achieve high accuracy.

The broader motivation remains relevant to healthy aging because mobility, activity, frailty, mood, sleep, chronic disease, and cognition are interdependent. A cross-sectional association between community-dwelling older adults with multimorbidity and physical activity level was graded, with higher physical activity levels associated with lower levels of frailty; the authors appropriately caution that a cross-sectional association does not establish prevention (24). The same distinction applies here: the pipelines classify observed data but do not demonstrate improvements in frailty, independence, cognition, or recovery.

Digital interventions can expand opportunities for activity, but effectiveness should not be inferred from technical performance alone. A 2026 meta-analysis of digitally delivered multimodal interventions using objective measurement found greater step counts per day, with low to very low evidence certainty, and moderate-to-vigorous activity, which was sensitive to publication bias (25). These findings support objective monitoring and human feedback while emphasizing that technology alone does not ensure sustained intensity, adherence, or functional change. A telerehabilitation platform should therefore be part of a care pathway in which professionals review, set goals, monitor progress, and escalate.

Acceptability is also crucial. In 2026, focus groups showed that older adults had good mobile-health literacy but believed using digital tools was less engaging, emotionally connected, or satisfying than face-to-face communication (26). Accordingly, even a high-performing classifier may have limited value if the interface is confusing, inaccessible, or perceived as a replacement for human care. The appropriate role for such tools is therefore to support routine measurement and to identify cases for clinician discussion, rather than to replace therapeutic relationships.

Recent interpretable screening studies similarly emphasize clear boundaries for use. Ye and Peng (23) built a model based on routine EHRs that focused on screening rather than diagnosis for their gradient boosting model. Zhang (27) combined accelerometry with neighborhood indicators for MoCA-defined impairment and framed the model as a prioritization tool rather than an etiologic inference. This distinction should be explicit in digital rehabilitation: model probabilities may support prioritization for review but do not establish etiology or determine treatment.

Key strengths include a reproducible workflow, a fixed random seed, automated data auditing, exact duplicate detection, multiple algorithms, stratified cross-validation, holdout evaluation, bootstrap confidence intervals, class-specific metrics, calibration, uncertainty analysis, feature family ablation, and model-agnostic feature importance. It was ensured that the two datasets were not concatenated and that a physical separation could be made between the exercise type and correct execution. Evidence is also limited, including poor performance in ablating motion and temporal aspects, confusion about the direction of knee extension, poor physical participant metadata, and an uncertain origin of the cognitive data.

These limitations are significant and narrow the range of inference. Firstly, both were retrospective secondary sources. The physical videos provided no participant or session identifiers, so we could not validate the subject or session groups. A random split of videos into training and test sets may place some videos from the same person, setting, or session in both sets. Exact duplicate removal can minimize, but not eliminate, literal duplication, and it does not eliminate near-duplicates or leakage from shared contexts. Thus, reported physical accuracy should not be generalized across subjects. Second, there is insufficient age, diagnosis, impairment, and therapist metadata from the physical dataset to make specific conclusions about older adults, stroke survivors, and frail patients.

Third, the physical sample is small: there are 315 videos and only 42-62 examples per class, so bootstrap intervals are wide, and class-specific estimates use only 8-12 test videos. Fourth, handcrafted features may overlook subtle differences in joint execution and may be affected by camera view, clothing, lighting, assistive devices, and occlusion. No external physical dataset was available for validation. Fifth, correctness labels were not based on therapist adjudication, a standard error rubric, or an interrater reliability assessment. The physical model thus creates labels based on the given dataset, not on clinically validated judgments of exercise appropriateness or safety.

Taken together, these limitations require conservative interpretation of the present findings. The available evidence supports a technical proof of concept for separate physical exercise-assessment and cognitive-status support components rather than evidence of individual recovery, patient-level multimodal integration, diagnosis, treatment effectiveness, or autonomous care. Generalizability should therefore not extend beyond the source datasets and the validation procedures used in this study. A future study should include a group of older adults who are already clinically characterized and obtain physical and cognitive assessments from the same individuals. Video validation should be organized by participant, site, and recording session, and include standardized therapist rubrics for execution quality, error type, level of therapist assistance, and safety. Before claiming generalizability, test the device in external settings, including various mobility aids, different devices, lighting, clothing, body size, and skin tone.

Future studies are needed to differentiate concurrent status classification and prediction of future decline and treatment response with regard to cognition. Assess the difference between a core model (without cognitive screening scores) and an augmented model (with MMSE/MoCA) at clinically relevant thresholds for calibration and net benefit. Missing data mechanisms, language, education, and cultural validity also need explicit assessment. Finally, evaluate clinical utility prospectively against usual care provided by clinicians, using functional outcomes, adherence, safety, patient experience, caregiver burden, and cost.

## 5 Conclusion

This study presents two independent, reproducible machine learning pipelines for rehabilitation exercise assessment and concurrent cognitive status classification. The physical random forest achieved 85.7% video-level accuracy and 84.9% macro F1 for the six-class exercise-by-correctness task; these estimates should not be interpreted as participant-independent validation because participant and session identifiers were unavailable. For cognitive status classification, Extra Trees achieved 93.6% accuracy, 92.3% macro F1, and AUC 0.978. Confidence-based review, feature importance, ablation, and class-specific error analysis provided information beyond overall accuracy.

These findings demonstrate technical feasibility within the source datasets, not clinical recovery, autonomous care, or patient-level multimodal integration. The physical participant demographics were insufficiently documented; participant- or session-level validation was unavailable; the correctness labels lacked independent therapist adjudication; and the provenance of the cognitive dataset and target construction were uncertain, with MMSE and GDS potentially target-proximal. The physical and cognitive datasets contained different, unlinked records. Prospective, participant-linked, clinically adjudicated validation is therefore required before clinical deployment; at present, the work should be viewed as a proof of concept for separate, clinician-supervised decision-support components.

## Data Availability

The datasets analyzed in this study are publicly available. The WLU Rehabilitation Posture dataset is available from Kaggle at: https://www.kaggle.com/datasets/sulaimanmuhammed/wlu-rehabilitation-posture. The Community Elderly Cognitive Health Dataset is available from Kaggle at: https://www.kaggle.com/datasets/ziya07/community-elderly-cognitive-health-dataset. Further inquiries can be directed to the corresponding author.
